# Dynamism under the hood: Coevolutionary maintenance of variation in hosts and parasites

**DOI:** 10.1371/journal.pbio.3004045

**Published:** 2026-10-23

**Authors:** Andrea L. Graham, David A. Chang van Oordt

**Affiliations:** 1 Department of Ecology & Evolutionary Biology, Princeton University, Princeton, New Jersey, United States of America; 2 Santa Fe Institute, Santa Fe, New Mexico, United States of America

## Abstract

Why do hosts and parasites vary so much? This Primer explores a new study in PLOS Biology revealing that infection outcomes strongly depend upon interacting effects of host and parasite genotypes, offering an evolutionary explanation for profound and persistent variability.

## Teaser

Why do hosts and parasites vary so much? A new study in *PLOS Biology* reveals that infection outcomes strongly depend upon interacting effects of host and parasite genotypes, offering an evolutionary explanation for profound and persistent variability.

Host defense capabilities are notoriously variable, as are parasite attack capabilities. Explaining why host and parasite populations are so heterogeneous is a key aim in both evolutionary biology and clinical medicine (e.g., [1]). A new study in *PLOS Biology* [2] sheds experimental light on evolutionary mechanisms that may sustain such variability. Using ambitious experiments supplemented with mathematical modeling, Belyi and colleagues [2] found that the fitness outcomes fueling natural selection on each partner depended upon which particular host and parasite genotypes were interacting. Such context-dependency of fitness is important evolutionarily because it means that no single host nor parasite genotype would dominate over time, which could in turn maintain genetic diversity in both populations.

Parasites, by definition, pose potent threats to host fitness. For example, a parasite infecting a susceptible host is likely to kill that host and/or block its reproduction, while a resistant host is likely to turn the tables, killing the parasite and/or blocking its reproduction. In each case, at the scale of the infected host, the outcome is potentially disastrous for one partner, glorious for the other. In a simple scenario, such effects would lead to the erosion of genetic variation at the population scale: e.g., universally susceptible host genotypes should disappear from the population via parasite-mediated directional selection against them. But in a scenario with coevolutionary countermoves—such as parasites evolving escape from particular resistance mechanisms, or hosts evolving novel resistance pathways—there may be no net direction of natural selection over time. Instead, antagonistic coevolution is predicted to generate balancing selection that fosters persistent genetic diversity at the population scale. Such expectations (cartooned in Fig 1) are grounded in theory (though contingent upon other factors such as geographical population structure [3]). Alas, empirical demonstrations are rare.

**Fig 1:**
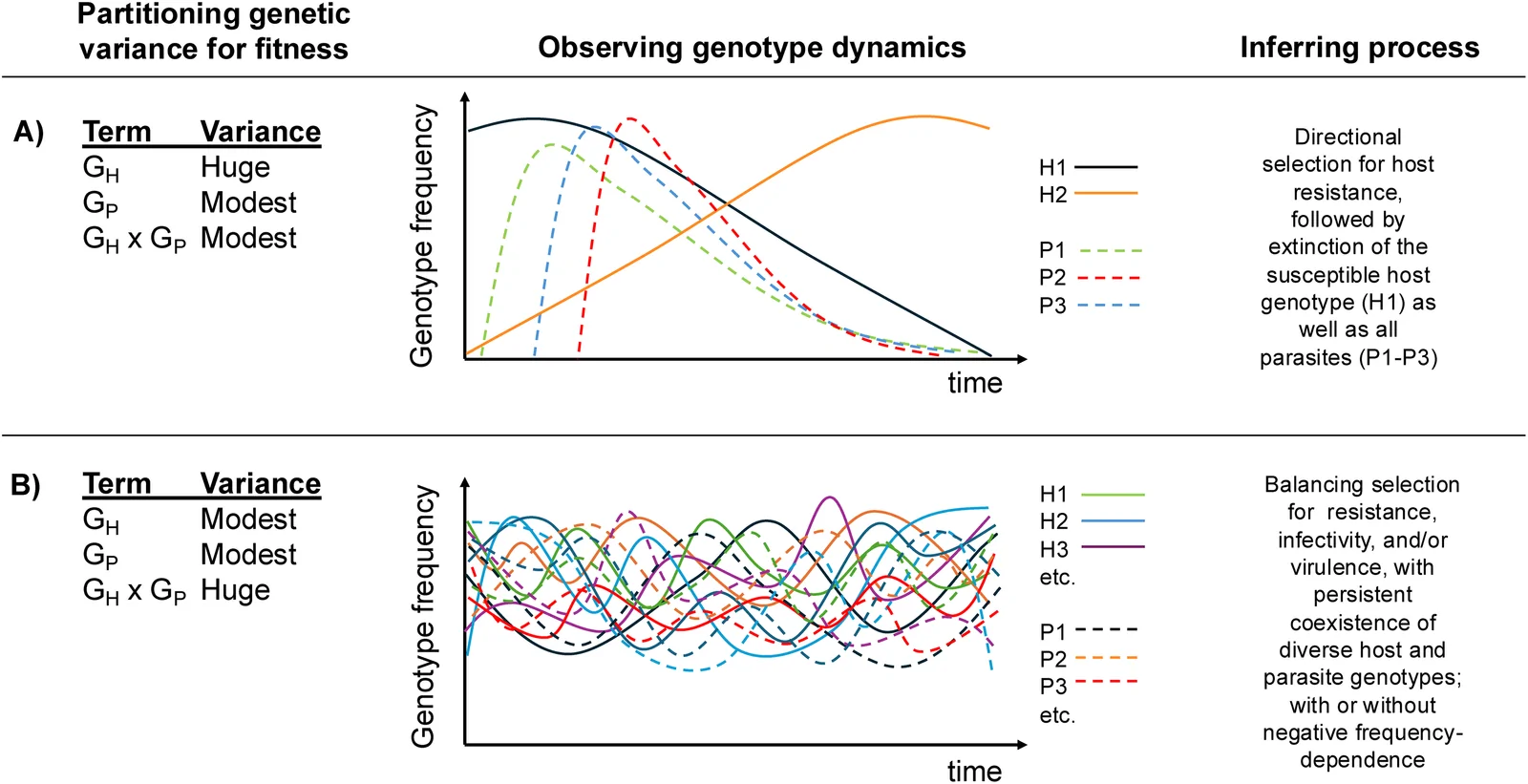
Differential partitioning of genetic variance for fitness predicts divergent genotype dynamics and evolutionary processes. A factorial experimental design that crosses multiple host genotypes against multiple parasite genotypes and measures fitness across all combinations enables statistical analysis of genetic variance for fitness, as in [2]. A key question is whether variance is explained primarily by main effects (host genotype G_H_ and/or parasite genotype G_P_) or by the interaction term, G_H_ x G_P_. In the example cartooned in **(A)**, fitness variance explained by G_H_>> both G_P_ and G_H_ x G_P_. Resulting genotype dynamics reflect a sustained fitness advantage for host genotype 2 (H2) over host genotype 1 (H1), no matter the parasite genotype (P1–P3). In this case, directional selection for host resistance followed by extinction of all parasites is inferred. By contrast, in the example cartooned in **(B)**, fitness variance explained by G_H_ x G_P_>> both G_H_ and G_P_. Resulting genotype dynamics reflect no sustained fitness advantage for any host (H1–7), nor any parasite (P1–P7). In this case, balancing selection is inferred, and all genotypes coexist over time. When the bulk of genetic variance for host and parasite fitness is in the interaction term (as in [2]), genetic variation can thus be maintained by host-parasite coevolution, which arguably exhibits great dynamism “under the hood” of sustained coexistence. [Please note: these cartoons represent just 2 illustrative examples of many possible outcomes; a subset of genotypes are identified in (B), to save space].

To investigate these possibilities, Belyi and colleagues studied genetic variance for fitness following exposure of fruit flies (*Drosophila melanogaster*) to vertically-transmitted sigma viruses [2]. They began by developing mathematical models to predict how additive and interactive genetic variance might emerge from coevolution of hosts with vertically-transmitted parasites. Their simulations predicted that antagonistic coevolution should erode additive genetic variance for fitness (i.e., a G_H_ main effect for host fitness and a G_P_ main effect for parasite fitness) and instead push fitness variance into the interaction term (G_H_ x G_P_). Belyi and colleagues then undertook a remarkable slate of experiments in which they pitted 9 fly genotypes against 10 virus genotypes, for a total of 90 replicated combinations [2]. For each, they measured key fitness-related traits such as viral transmission (i.e., rate of vertical transmission from infected parents to offspring), viral load, and virulence (i.e., negative effects on fly fecundity and hatch rate). In line with simulation results, their data analyses revealed that the bulk of the genetic variance in these traits was explained by G_H_ x G_P_ interaction terms rather than main effects of fly or virus genotype. They therefore found that there was no one fly genotype that was universally resistant, nor one universally transmissible or virulent viral genotype. These findings are more consistent with the balancing selection scenario of Fig 1B than with the directional selection scenario of Fig 1A.

Fascinatingly, the experiments included fly genotypes harboring polymorphisms known to severely restrict sigma virus replication, yet even those did not offer general resistance: instead, they conferred resistance against only a subset of viral genotypes, and thus were caught up in the G_H_ x G_P_ complexities. Sex effects also manifested primarily within the interaction term, suggesting that female and male hosts experience differential G_H_ x G_P_ dependence of fitness [2]. Biology may not be simple, but at least it is interesting!

The authors conclude that because most of the observed genetic variance was in the G_H_ x G_P_ interaction term, such variance remains “cryptic,” with coevolution “masking” heritable variation from directional selection [2]. They also posit that their results are consistent with negative frequency-dependent selection (NFDS), which promotes diversification by favoring rare variants until they become common (i.e., via rare advantage). Indeed, their mathematical model revealed some parameter-dependent persistent cycling [2] consistent with general theory on NFDS [4]. It is thus possible that time-lagged NFDS, as predicted under the Red Queen model of antagonistic coevolution [5], may underlie dynamics such as those depicted in Fig 1B. Yet there are multiple mechanisms of balancing selection, including NFDS, heterozygote advantage, and spatiotemporal fluctuating selection, among others, all of which can lead to persistence of genetic variation [4,6]. Belyi and colleagues thus leave the important question of the mechanism of balancing selection open, as they have yet to demonstrate rare advantage empirically and confirm that NFDS is at work in the fruit fly-sigma virus system.

Empirical demonstration of NFDS is not trivial, however, especially in natural host-parasite systems. G_H_ x G_P_ is necessary but not sufficient: at a minimum, it also requires time series data on genotypes and parasite burdens from natural populations (e.g., suggesting rare advantage in bank voles fighting newly invasive parasites [7]). A truly state-of-the-art demonstration of NFDS requires time series data on genotypes and fitness on both microevolutionary and macroevolutionary time scales [5] and/or time-shift experiments, in which hosts are exposed to parasites of the past, present, and future [8]. Regardless of the mechanism of balancing selection, and even when static snapshots of variance appear “cryptic,” the inherent dynamism of host-parasite coevolution (Fig 1B) may be maintaining host and parasite genetic diversity into the future.

Belyi and colleagues have thereby set a high bar for linked empirical and theoretical investigation of the evolutionary maintenance of genetic diversity among hosts and parasites. Tractability of their empirical system is inarguable, so many avenues of research remain open to them. What future directions might they pursue? Beyond investigation of NFDS as outlined above, exploration of the evolution of known fly resistance alleles in the face of sigma virus diversity could be a rich vein of research. Indeed, the data collected by Belyi and colleagues highlight the critical importance of using multiple genotypes in experiments whenever possible, to identify susceptibility, infectivity, or restriction factors that might be missed if only a single genotype is used. The findings are likely to also inspire plenty of new work by others. Perhaps we will see new experiments in other systems amenable to dissection of the causes of persistent variation in host and parasite populations over even deep time? Perhaps we will see investigation of the directions and impact of sex x G_H_ x G_P_ effects across diverse empirical systems, including those such as pipefish, in which both sex roles and immunological correlates of pregnancy are reversed [9]? There is no doubt a very great deal going on “under the hood” in the dynamics of hosts and parasites in natural populations, but further work is needed to unpick what evolutionary processes are underway and how generally they may apply across organismal diversity.
